# Sunspot data and human longevity

**DOI:** 10.1016/j.dib.2018.10.168

**Published:** 2018-11-06

**Authors:** George E. Davis, Walter E. Lowell

**Affiliations:** Psybernetics Research Group, 28 Eastern Ave., Augusta, Maine 04330, USA

**Keywords:** Ultraviolet radiation, Human lifespan, Sunspot number, Solar energy, Multiple sclerosis, Epigenetics

## Abstract

Solar energy at birth and human lifespan, *Journal of Photochemistry & Photobiology B* 186 (2018)59–68.

This paper uses National Center for Health Statistics (NCHS) death data collected from 1979- 2013, inclusive, and average monthly solar intensity as measured by sunspot number collected from the National Oceanic and Atmospheric Administration (NOAA) from 1900–2013, inclusive.

**Specifications table**

**Table:** 

Subject area	*Physics, Biology, Medical*
More specific subject area	*Solar energy, Epigenetics, and lifespan*
Type of data	*Raw Data, Table, text file, graph, figure*
How data was acquired	*Requested from National Center for Health Statistics (NCHS), National Oceanic and Atmospheric Administration(NOAA)*
Data format	*Raw, filtered,*
Experimental factors	*Dependent variable: Age at Death, Independent variables: SSN, sex, race*
Experimental features	*Cross sectional study*
Data source location	*USA National Center for Health Statistics (NCHS), National Oceanic and Atmospheric Administration(NOAA)*
Data accessibility	*Data used with this article is in public repository and available*
Related research article	George E. Davis, Jr., Walter E. Lowell
	*Sunspot data and human longevity*
	Davis GE & Lowell WE*, Journal of Photochemistry & Photobiology B* 186 (2018) 59–68.

**Value of the data**.

The data used in this article are freely available to the public:

• Death data can be used to assess lifespan by cause of death and sunspot data can be used to track solar cycles as they pertain to lifespan.
• These data may allow researchers to see how levels of ultraviolet radiation (UVR) energy may affect lifespan by using animal models.
• These data might be used prospectively to determine the effects of solar energy on specific causes of death.

## 1 External Data

### 1.1 For death data

https://www.cdc.gov/nchs/nvss/deaths.htm A specific request is required for deidentified data or specific formats [1]. For solar data: www.noaa.gov [2] Psybernetics Research Group has collected average monthly ground UVR data (UV-A and UV-B as well as total solar energy, both in joules/m^2^, from 2007 through 2017 in Augusta, ME [latitude +44.308992 degrees; longitude −69.769008 degrees]). Data from this period, largely including Solar Cycle 24, is available upon request from georgedavi@gmail.com [3].

#### 1.1.1 NCHS death record formats

Alphabetic List of Variables and Attributes.

# Variable Type Len Format Informat:

6 cause Char 4.

7 cause72 Num 8.

13 cause113 Num 8.

8 datayear Num 8.

11 fipstres Char 2.

12 hisprace Num 8.

4 mo_brth Num 8.

2 mo_dth Num 8.

9 racer5 Num 8.

1 resstat Num 8.

3 sex Num 8 BEST12. F12.

5 st_brth Char 2 $2. $2.

10 yr_brth Num 8.

## 2 Data

The data shared here include sunspot number (SSN) by year and month from 1900 to 2009, statistical tables from the original data sets and lifespan plots of data for various age groups. NCHS data formats are described in this attachment.

### 2.1 Cohort data

78,645,528 death records were obtained from NCHS 1979 to 2013. The following variables used from the dataset: year of birth (YOB), month of birth (MOB), sex, year of death (YOD) and race (White, Black, Native-American, and Asian). The dependent variable was lifespan, calculated as the YOB minus the YOD. Records with a lifespan longer than 113 years were designated as outliers and deleted from the analysis. Birth years originally ranged from 1866 to 2013. Table 1 (in the original paper) summarizes the original and scrubbed cohort data by sample size, mean age, sex, and race. For this analysis, deaths that occurred by accidents, suicides and war casualties were deleted as well as restricting the cohort to birthdates from 1900 to 2013. Suicides were deleted as their number was very small relative to the entire dataset. The final dataset was comprised 31,807,486 females and 31,947,344 males (total=63,7543,830). Multiple sclerosis data (*N*=85,202) was derived from the entire dataset by diagnosis code (ICD 10=G35, ICD 9=340).

### 2.2 Solar data

Solar cycle data as measured by monthly SSN was collected and used as a surrogate for UVR; for example, the higher the SSN the greater the UVR intensity. The average number of annual sunspots per month and per year was collected from NOAA web site: (https://www.ngdc.noaa.gov/stp/space-weather/solar-data/solar-indices/sunspot-numbers) or see Appendix B for the SSN data). (2) To examine the influence of solar radiation on lifespan, sunspot numbers by year and month were matched-merged by year and month with each cohort case׳s birth year and birth month. Mean SSN for the entire cohort was 47.68 (41.57 median) with a minimum of 0 and maximum of 253 and for the scrubbed data used in this analysis the mean SSN was 43.4 (38.1 median) with a minimum of 0 and a maximum of 253.

## 3 Experimental design, material and methods

### 3.1 Analysis strategy

To examine the influence of solar radiation on lifespan, sunspot numbers by year and month were matched-merged by year and month with each cohort case׳s birth year and birth month.

Two strategies were used for the analysis. The first was to use regression analysis (SAS 9.3) to test the hypothesis that UVR, as measured by SSN at year of birth (YOB) and month of birth (MOB), affects subsequent age at death; for example, lifespan. Table 2 (in the main manuscript) displays the correlation matrix for these variables. The regression (GLM) model tested included the relationship between lifespan, SSN, sex and their respective interactions.

The second strategy was to plot lifespan by SSN to visually assess the relationship between increasing SSN (UVR at the time of birth) and lifespan by sex for all races. Charts were created based on summarizing data by categorizing SSN into intervals of 10 starting with 0–10, 10–20, 20–30, etc. The mean SSN and mean lifespan with respective standard deviations were calculated. Table 3 (in the main manuscript) displays a typical table for White males showing SSN interval, mean and standard deviation for SSN, for mean age by sunspot grouping, and group sample size. The mean lifespan by sex for the White and Black races was plotted by SSN and can be found in Figures 3 and 4 (in the main manuscript). For those who are interested, except for the 90–113 years old cohort (Figure 7 in the manuscript), all plots by SSN group by age group; e.g., infancy, early life, puberty and post-menopause are in Appendices A-1 to A-4, Appendices A-1 to A-4, Appendices A-1 to A-4, Appendices A-1 to A-4; a table of the average lifespan for each of each of these groups is in Appendix A-5. Appendices A-6 to A-11, Appendices A-6 to A-11, Appendices A-6 to A-11, Appendices A-6 to A-11, Appendices A-6 to A-11, Appendices A-6 to A-11 display additional SAS statistical tables referring to the data used in the original manuscript and may be of interest to statisticians.

## Figures and Tables

**Table A5:** Average lifespan for various age groups.

**Age group (range in years)**	**Grp 1 male average Age (years) [SD]**	**Grp 1 female average Age (years) [SD]**	**N group 1 male** (M/F ratio) **N group 1 female**	**SSN group at lifespan decrease** males:females:
**0–2**	5.0563	5.2333	326,448	170–180
	(months)	(months)	(1.31)	170–180
	[0.198]	[0.154]	249,106	
				
**1–3**	1.5045	1.4958	172,856	170–180
	[0.033]	[0.026]	(1.30)	170–180
			133,178	
				
**11–14**	12.6872	12.6254	71,081	100–110
	[0.031]	[0.017]	(1.65)	100–110
			43,046	
				
**25–28**	26.5213	26.5695	420,065	No decrease
	[0.013]	[0.010]	(2.76)	For either gender
			152,469	
				
**35–40**	37.6529	37.7252	754,076	80–90
	[0.093]	[0.084]	(1.85)	90–100
			408,227	
				
**45–50**	47.6694	47.7032	1,544,464	90–100
	[0.018]	[0.017]	(1.69)	Biphasic
			912,700	90–100
				
**55–60**	57.2757	57.2999	2,909,397	90–100
	[0.374]	[0.376]	(1.62)	90–100
			1,811,751	
				
**60–65**	63.0497	63.0710	3,302,137	100–110
	[0.045]	[0.032]	(1.52)	100–110
			2,173,784	
				
**75–85**	77.3932	80.0043	11,127,219	100–110
	[0.690]	[0.478]	(0.96)	100–110
			11,550,651	
				
**90–100**	92.9105	93.5647	2,937,037	70–80
	[0.202]	[0.341]	(0.41)	70–80
			7,103,852	
			Male sum:	
			23,564,780	
			(49%)	
			Female sum:	
			24,538,764	
			(51%)	

**Table:** 

rgrp	N	Mean	Std Dev	Std Err	Minimum	Maximum
GE 90	6379227	128.4	32.0999	0.0127	90.1000	253.8
LT90	33576792	35.2228	25.9797	0.00448	0	89.9000
Diff (1–2)		93.1301	27.0499	0.0117		

rgrp	Method	Mean	95% CL Mean		Std Dev	95% CL Std Dev	
GE 90		128.4	128.3	128.4	32.0999	32.0823	32.1176
LT90		35.2228	35.2140	35.2316	25.9797	25.9735	25.9859
Diff (1–2)	Pooled	93.1301	93.1072	93.1530	27.0499	27.0440	27.0558
Diff (1–2)	Satterthwaite	93.1301	93.1037	93.1565			

Method	Variances	DF	t Value	Pr>|t|
Pooled	Equal	4E7	7971.44	<.0001
Satterthwaite	Unequal	8.04E6	6910.35	<.0001

Equality of Variances				
Method	Num DF	Den DF	F Value	Pr>F
Folded F	6.38E6	3.36E7	1.53	<.0001

**Table:** 

Moments			
N	4606983	Sum Weights	4606983
Mean	60.7371073	Sum Observations	279814821
Std Deviation	23.1394675	Variance	535.434955
Skewness	−0.7824813	Kurtosis	0.34884685
Uncorrected SS	1.94619E10	Corrected SS	2466739202
Coeff Variation	38.0977437	Std Error Mean	0.01078065

Basic statistical measures			
Location		Variability	
Mean	60.73711	Std deviation	23.13947
Median	63.00000	Variance	535.43496
Mode	0.00000	Range	115.00000
		Interquartile range	29.00000

Tests for location: Mu0=0				
Test	Statistic		*p* value	
Student׳s *t*	t	5633.899	Pr > |t|	<0.0001
Sign	M	2225433	Pr >= |M|	<0.0001
Signed rank	S	4.953E12	Pr >= |S|	<0.0001

**Table:** 

NCHS data 1979 to 2013
dataset data.all79_13
if ageatdeath >115 then delete
data.all79_13 ALL Races all ages 79 to 13 data
All data - Distribution statistics for MALES
SSN LT 90 & GE 90

**Table:** 

Moments			
N	6379227	Sum weights	6379227
Mean	54.0152072	Sum observations	344575268
Std deviation	21.7242974	Variance	471.945099
Skewness	-0.5491325	Kurtosis	−0.0249084
Uncorrected SS	2.16229E10	Corrected SS	3010644447
Coeff variation	40.2188542	Std error mean	0.00860125

Basic statistical measures			
Location		Variability	
Mean	54.01521	Std deviation	21.72430
Median	56.00000	Variance	471.94510
Mode	0.00000	Range	115.00000
		Interquartile range	28.00000

Tests for location: Mu0=0				
Test	Statistic		*p* value	
Student׳s *t*	t	6279.923	Pr > |t|	<0.0001
Sign	M	3088380	Pr >= |M|	<0.0001
Signed rank	S	9.538E12	Pr >= |S|	<0.0001

**Table:** 

rgrp	N	Mean	Std Dev	Std Err	Minimum	Maximum

GE 90	6379227	54.0152	21.7243	0.00860	0	115.0
LT90	33576811	70.5578	18.1468	0.00313	0	115.0
Diff (1–2)		−16.5426	18.7638	0.00810		

rgrp	Method	Mean	95% CL mean		Std dev	95% CL Std dev	
GE 90		54.0152	53.9983	54.0321	21.7243	21.7124	21.7362
LT90		70.5578	70.5517	70.5640	18.1468	18.1425	18.1512
Diff (1–2)	Pooled	−16.5426	−16.5585	−16.5268	18.7638	18.7597	18.7679
Diff (1–2)	Satterthwaite	−16.5426	−16.5606	−16.5247			

Method	Variances	DF	*t* Value	Pr>|t|
Pooled	Equal	4E7	−2041.3	<.0001
Satterthwaite	Unequal	8.16E6	−1807.2	<.0001

Equality of variances				
Method	Num DF	Den DF	F Value	Pr>F
Folded F	6.38E6	3.36E7	1.43	<.0001

**Table:** 

NCHS data 1979 to 2013
dataset data.all79_13
if ageatdeath>115 then delete
data.all79_13 ALL Races all ages 79 to 13 data
All data - Distribution statistics for FEMALES
SSN LT 90 & GE 90

**Table:** 

Moments			
N	34083435	Sum weights	34083435
Mean	77.3822772	Sum observations	2637453815
Std Deviation	16.5075125	Variance	272.497968
Skewness	−1.8429927	Kurtosis	5.12071072
Uncorrected SS	2.1338E11	Corrected SS	9287666492
Coeff Variation	21.3324201	Std error mean	0.00282755

Basic statistical measures			
Location		Variability	
Mean	77.38228	Std deviation	16.50751
Median	81.00000	Variance	272.49797
Mode	86.00000	Range	115.00000
		Interquartile range	17.00000

Tests for location: Mu0=0				
Test	Statistic		*p* value	
Student׳s *t*	t	27367.27	Pr>|t|	<0.0001
Sign	M	16905293	Pr>=|M|	<0.0001
Signed rank	S	2.858E14	Pr>= |S|	<0.0001

**Table:** 

NCHS data 1979 to 2013
dataset data.all79_13
if ageatdeath>115 then delete
data.all79_13 ALL Races all ages 79 to 13 data
All data - Distribution statistics for MALES
SSN LT 90 & GE 90

**Table:** 

Moments			
N	33576811	Sum weights	33576811
Mean	70.5578454	Sum observations	2369107439
Std Deviation	18.1468249	Variance	329.307253
Skewness	−1.4690249	Kurtosis	2.73276925
Uncorrected SS	1.78216E11	Corrected SS	1.10571E10
Coeff Variation	25.7190746	Std error mean	0.00313171

Basic statistical measures			
Location		Variability	
Mean	70.55785	Std deviation	18.14682
Median	74.00000	Variance	329.30725
Mode	79.00000	Range	115.00000
		Interquartile range	20.00000

Tests for location: Mu0=0				
Test	Statistic		*p* value	
Student׳s *t*	t	22530.17	Pr>|t|	<0.0001
Sign	M	16612798	Pr>=|M|	<0.0001
Signed Rank	S	2.76E14	Pr>=|S|	<0.0001

## Appendix A-1

Male and Female Infants who lived an average of 5 months by Sunspot Number Group.

## Appendix A-2

Males and Females who lived from 1–3 years by Sunspot Number Group.

## Appendix A-3

Males and Females who lived from 11–14 years by Sunspot Number Group.

## Appendix A-4

Males and Females who lived from 55–60 years old by Sunspot Number Group.

## Appendix A-5

See Table A5.

## Appendix A-6

Variable: SSN (SSN)

## Appendix A-7

The UNIVARIATE Procedure Variable: ageatdeath rgrp=GE 90

## Appendix A-8

The UNIVARIATE Procedure Variable: ageatdeath rgrp=GE 90

## Appendix A-9

The TTEST Procedure Variable: ageatdeath sex=1

## Appendix A-10

The UNIVARIATE Procedure Variable: ageatdeath rgrp=LT90

## Appendix A-11

The UNIVARIATE Procedure Variable: ageatdeath rgrp=LT90

## Appendix B

See Excel file for SSN data.
