# Supplementary material for: Sunspot data and human longevity
Source: Data Brief. 2018 Nov 6;21:1579–90. doi: 10.1016/j.dib.2018.10.168 (PMC6240642; doi:10.1016/j.dib.2018.10.168)
Supplement: Supplementary file 1 — Supplementary material [file mmc1.docx]

**DECLARATIONS:**

*Ethics approval and consent to participate:* Only de-identified data was obtained from the NCHS. No specific human or animal was used for this study.

*Consent for publication:* Both authors (GED and WEL) give consent for publication.

*Availability of data and material:* NCHS data are publicly available upon request. NOAA data (see Data in Brief) are available on website <http://www.noaa.gov>. Any data used in the Figures are available upon request.

*Competing interests:* The authors are not aware of any competing interests.

*Funding:* No outside funding or grants were used for this work.

*Authors’ contributions:* Both authors were involved writing the manuscript. WEL did the statistical work in the statistical package SAS. GED was responsible for the Figures in the study.
